# A fresh look at the evolution and diversification of photochemical reaction centers

**DOI:** 10.1007/s11120-014-0065-x

**Published:** 2014-12-16

**Authors:** Tanai Cardona

**Affiliations:** Department of Life Sciences, Imperial College London, Exhibition Road, London, SW7 2AZ UK

**Keywords:** Cyanobacteria, Acidobacteria, Chloroflexi, Heliobacteria, Chlorobi, Photosystem

## Abstract

In this review, I reexamine the origin and diversification of photochemical reaction centers based on the known phylogenetic relations of the core subunits, and with the aid of sequence and structural alignments. I show, for example, that the protein folds at the C-terminus of the D1 and D2 subunits of Photosystem II, which are essential for the coordination of the water-oxidizing complex, were already in place in the most ancestral Type II reaction center subunit. I then evaluate the evolution of reaction centers in the context of the rise and expansion of the different groups of bacteria based on recent large-scale phylogenetic analyses. I find that the Heliobacteriaceae family of Firmicutes appears to be the earliest branching of the known groups of phototrophic bacteria; however, the origin of photochemical reaction centers and chlorophyll synthesis cannot be placed in this group. Moreover, it becomes evident that the Acidobacteria and the Proteobacteria shared a more recent common phototrophic ancestor, and this is also likely for the Chloroflexi and the Cyanobacteria. Finally, I argue that the discrepancies among the phylogenies of the reaction center proteins, chlorophyll synthesis enzymes, and the species tree of bacteria are best explained if both types of photochemical reaction centers evolved before the diversification of the known phyla of phototrophic bacteria. The primordial phototrophic ancestor must have had both Type I and Type II reaction centers.

## Phototrophic bacteria and their reaction centers

Understanding the origin, evolution, and diversification of bacteriochlorophyll- and chlorophyll-based phototrophy has been a difficult challenge. A major barrier in our understanding derives from the scattered placement of phototrophic bacteria in the tree of life, summed to a lack of consensus regarding the evolutionary relationships of the different bacterial groups (Woese 1987; Doolittle 1999; Brown et al. 2001; Ciccarelli et al. 2006; Battistuzzi and Hedges 2009). Moreover, different components of the photosynthetic process seem to have followed independent evolutionary pathways (Blankenship 1992; Xiong and Bauer 2002; Mix et al. 2005; Hohmann-Marriott and Blankenship 2011; Sousa et al. 2013). In particular, the origin of water oxidation by Photosystem II still remains one of the greatest mysteries in the evolution of life. Only by understanding the evolutionary relationships among all phototrophic bacteria within a solid framework for the diversification of prokaryotes, it will be possible to reconstruct an unequivocal scenario for the molecular evolution of photosynthesis. In this review, I reevaluate the evolutionary relationships of Type I and Type II reaction centers based on their phylogeny. I then frame the evolution of reaction centers in the context of the evolution and diversification of bacteria based on recent and comprehensive studies. I conclude that the origin of two distinct types of reaction centers predates the rise and expansion of the major phyla of bacteria and therefore should not be placed in any of the known groups of phototrophs.

### Seven phyla

Bacteria with photochemical reaction centers are currently distributed in seven different phyla (see Table 1). These are: Chloroflexi, Chlorobi, Firmicutes, Proteobacteria, Cyanobacteria, and the recently discovered Acidobacteria (Bryant et al. 2007) and Gemmatimonadetes (Zeng et al. 2014). All of these phyla contain phototrophic and non-phototrophic clades. The phylum Chloroflexi is thought to be subdivided into at least seven different classes (Yamada et al. 2006; Yarza et al. 2010; Loffler et al. 2013; Kawaichi et al. 2013), with the classes Chloroflexi and Anaerolineae having phototrophic representatives (Klatt et al. 2011). The phylum Chlorobi was thought to be composed only of phototrophic bacteria (Bryant and Liu 2013), but recent discoveries have expanded the diversity of the phylum to include non-phototrophic representatives (Iino et al. 2010). The phototrophic Chlorobi are now grouped in the order Chlorobiales within the class Chlorobea (Iino et al. 2010). The phylum Firmicutes is mostly composed of organisms incapable of phototrophy, and only the heliobacteria are known to have photochemical reaction centers (Sattley et al. 2014). They are grouped as the family Heliobacteriaceae within the class Clostridia (Madigan 2006; Madigan et al. 2010). The phylum Proteobacteria is subdivided into six classes: the Alpha-, Beta-, Gamma-, Delta-, Epsilon-, and Zetaproteobacteria. Phototrophic proteobacteria are only found within the classes Alpha-, Beta-, and Gammaproteobacteria. The phylum Acidobacteria has only one described species with photochemical reaction centers (Bryant et al. 2007), *Candidatus* *Chloracidobacterium thermophilum*, hereafter referred as *C. thermophilum*. Although the phylum Acidobacteria is very diverse and of ecological importance, it remains poorly studied and underrepresented in culture collections (Barns et al. 1999, 2007; Ward et al. 2009).

**Table 1:** Phototrophic groups, reaction center types, and pigment forms

Phylum	Phototrophic taxons	Reaction center type	Oligomericity	Reaction center subunit name	Chlorophyll type
Firmicutes	(Family) Heliobacteriaceae	I	Homodimer	PshA	Bchl *g*
Chlorobi	(Class) Chlorobea	I	Homodimer	PscA	Bchl *a*, *c*, *d*, *e*
Acidobacteria	(Species) *Chloracidobacterium thermophilum* ^a^	I	Homodimer	PscA	Bchl *a*, *c*
Chloroflexi	(Class) Anaerolineae Chloroflexia	II	Heterodimer	L (PufL), M (PufM)	Bchl *a*, *c*
Proteobacteria	(Class) Alphaproteobacteria Betaproteobacteria Gammaproteobacteria	II	Heterodimer	L, M	Bchl *a*, *b*
Gemmatimonadetes	(Species) *Gemmatimonas* sp. AP64^a^	II	Heterodimer	L, M	Bchl *a*
Cyanobacteria	(Class) Oxyphotobacteria^b^	I	Heterodimer	PsaA, PsaB	Chl *a*, *b*, *d*, *f* ^c^
		II	Heterodimer	D1 (PsbA), D2 (PsbD)	

^a^Only one phototrophic species in this phylum has been described to date

^b^Newly proposed class name based on the discovery of the Melainabacteria

^c^The type of chlorophyll used depends on the species of Cyanobacteria and/or environmental conditions

The phylum Cyanobacteria was thought to be unique because, until just a few years ago, all described species were capable of oxygenic photosynthesis. However, an uncultured diazotrophic cyanobacterium, *Candidatus Atelocyanobacterium thalassa*, was shown to have lost Photosystem II, carbon fixation, and other metabolic capabilities (Zehr et al. 2008; Thompson et al. 2012; Hagino et al. 2013). Thompson et al. (2012) demonstrated that it is the symbiotic partner of a eukaryotic alga and still retains Photosystem I to power nitrogenase. Cyanobacteria are classified in five ‘sections’ based on morphology and their mode of cell division (Rippka et al. 1979), but phylogenetic analyses showed that only heterocystous Cyanobacteria are monophyletic. Today, a consensus regarding the classification of the phylum Cyanobacteria is lacking, but 16S rRNA (Turner et al. 1999; Honda et al. 1999) and recent genomic studies (Shih et al. 2013) point to the existence of seven to ten clades of oxygenic phototrophs. A major leap in Cyanobacteria genomics occurred last year, after the sequencing of almost sixty new genomes encompassing the full diversity of the phylum (Dagan et al. 2013; Shih et al. 2013), including a new *Gloeobacter* genome (Saw et al. 2013).

A non-photosynthetic clade of bacteria closely related to the phylum Cyanobacteria was discovered recently and named the Melainabacteria (Di Rienzi et al. 2013). This group of bacteria is commonly found in the mammalian gut, although free-living representatives have also been discovered. Melainabacteria lack reaction centers, chlorophyll synthesis, and the ability to grow autotrophically. In a later study, Soo et al. (2014) proposed that the Melainabacteria should be classified as a new class of non-photosynthetic Cyanobacteria.

The newest addition to the list of phyla with phototrophic bacteria is the Gemmatimonadetes (Zeng et al. 2014). This phylum has only a couple of cultured strains, yet members of this group are widespread in nature (Zhang et al. 2003; Zeng et al. 2014). Zeng et al. (2014) showed that *Gemmatimonas* sp. AP64 has a full complement of bacteriochlorophyll synthesis and reaction center genes, which were acquired by horizontal gene transfer from a phototrophic proteobacterium, possibly of the Gammaproteobacteria. It should be expected that as the diversity of bacteria is mapped out at a genomic level, novel phototrophic bacteria will continue to be discovered.

### Two types of reaction centers

**Fig. 1:**
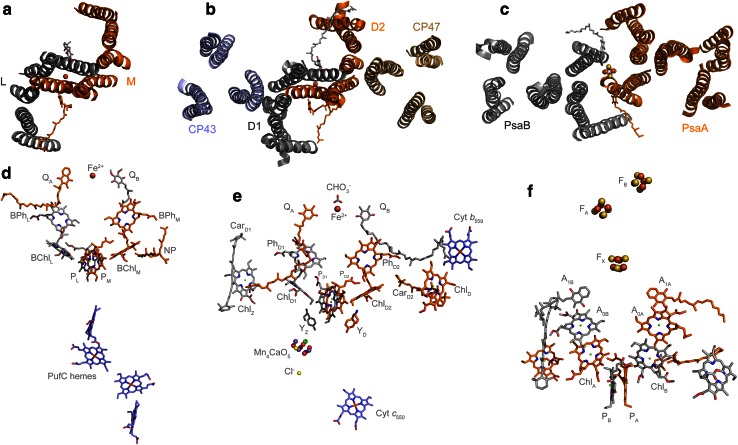
Reaction center architecture and cofactors. **a** Top view of a Type II reaction center from *Blastochloris viridis* highlighting the position of the transmembrane helices, PDB ID: 2PRC (Lancaster and Michel 1997). **b** Top view of Photosystem II from *Thermosynechococcus vulcanus* where only the transmembrane helices from the reaction center subunits (D1 and D2) and antenna proteins (CP43 and CP47) are highlighted, PDB ID: 3ARC (Umena et al. 2011). **c** Top view of Photosystem I from *Synechococcus elongatus*, PDB ID: 1JB0 (Jordan et al. 2001). **d** Cofactors in the Type II reaction center from *B. viridis*, the pigments colored in *orange* are coordinated or held by the M subunit, while those in *gray* by the L subunit. **e** Cofactors in Photosystem II, only those held by the D1 (*gray*) and D2 (*orange*) proteins are shown. **f** Cofactors in Photosystem I, those coordinated by the PsaA protein are colored in *orange*, and those by the PsaB are colored in *gray*. Besides the main pigments involved in charge separation, there are remarkable similarities in the position of certain accessory carotenoids and chlorophylls between Photosystem II (Car_D1_, Car_D2_, Chl_Z_, Chl_D_) and Photosystem I, suggesting that these may have been present in the ancestral reaction center

Photochemical reaction centers exist in two basic forms differentiated by some structural features and the nature of the terminal electron acceptor (Fig. 1). Type II reaction centers are found in the phototrophic members of the phylum Chloroflexi, Proteobacteria, and Gemmatimonadetes. Homodimeric Type I reaction centers are found in heliobacteria, Chlorobiales, and *C. thermophilum*. Cyanobacteria are the only group of photosynthetic bacteria known to carry both types of reaction centers (Govindjee and Shevela 2011): Photosystem II, the water-oxidizing enzyme, and a heterodimeric Type I reaction center, Photosystem I.

All known Type II reaction centers are heterodimeric, where the redox cofactors involved in photochemistry are held by two different transmembrane proteins, PufL (L) and PufM (M) in anoxygenic Type II reaction centers (Proteobacteria, Chloroflexi, and Gemmatimonadetes), and PsbA (D1) and PsbD (D2) in Photosystem II (Cyanobacteria). They all have in common the presence of (bacterio)pheophytin as a primary electron acceptor, a bound quinone (*Q*_A_), and a mobile quinone (*Q*_B_) as the terminal electron acceptor. *Q*_B_ is exchangeable with the membrane pool upon reduction. In addition, they are also characterized by a non-heme Fe^2+^ coordinated by two pairs of histidines from each reaction center subunit, bridging *Q*_A_ with *Q*_B_ (Cardona et al. 2012) (see Fig. 1).

**Fig. 2:**
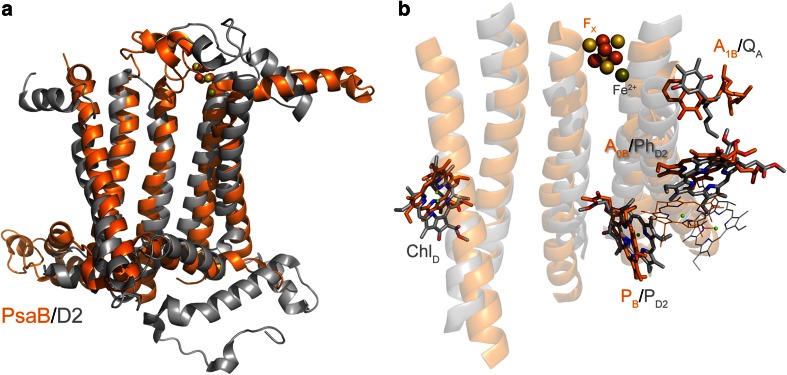
Comparison of a Type II reaction center subunit, D2 of Cyanobacterial Photosystem II (*gray*), and a Type I reaction center subunit (last five transmembrane helices) of the PsaB protein of Photosystem I (*orange*). **a** Overlap of D2 and PsaB; the *F*_X_ cofactor from Photosystem I and the non-heme Fe^2+^ from Photosystem II have been displayed as spheres to show their relatives positions. **b** Overlap of some of the cofactors coordinated by D2 (*gray*) and PsaB (*orange*). The peripheral chlorophylls, Chl_Z_ and Chl_D_, are conserved in these two subunits (Baymann et al. 2001). This histidine is also found in the sequences of all Type I reaction centers, with the exception of *C. thermophilum* and the anoxygenic Type II reaction centers. Its presence in Photosystem II implies that it was present in the most ancestral reaction center. The position of some of the chlorophylls, the quinones, *F*_X_, and non-heme Fe^2+^ is also very similar, yet the mode of coordination varies depending on the type of reaction center. The alignment of the subunits was made with the CEalign (Jia et al. 2004) plugging of Pymol (Molecular Graphics System, Version 1.5.0.4 Schrödinger, LLC)

Type I reaction centers are found as homodimers in anoxygenic phototrophic bacteria [e.g., Chlorobiales (Buttner et al. 1992), heliobacteria (Liebl et al. 1993), and *C. thermophilum* (Bryant et al. 2007)] and as heterodimers in members of the Cyanobacteria (Fish et al. 1985; Cantrell and Bryant 1987). In heliobacteria, the reaction center protein is named PshA, and the homologous proteins in the Chlorobiales and *C. thermophilum* are named PscA (Bryant 1994). In the phylum Cyanobacteria, the reaction center subunits are known as PsaA and PsaB. Type I reaction center proteins can be considered as having two domains: an antenna domain at the N-terminus encompassing the first six transmembrane helices, and a reaction center domain located at the C-terminus encompassing the last five transmembrane helices. The antenna domain has structural homology to the CP43 and CP47 subunits of Photosystem II (Fig. 1) (Vermaas 1994; Rutherford et al. 1996; Rutherford and Nitschke 1996; Mix et al. 2004; Vasil’ev and Bruce 2004), and the reaction center domain has structural homology to the core subunits of Type II reaction centers, L, M, D1, and D2 (Nitschke and Rutherford 1991; Fromme et al. 1996; Schubert et al. 1998; Baymann et al. 2001; Sadekar et al. 2006), see Fig. 2. Type I reaction centers are also characterized by having as terminal electron acceptors three consecutive Fe_4_S_4_ clusters, *F*_X_, *F*_A_, and *F*_B_ (Fig. 1). *F*_X_ is coordinated by two cysteine residues from each reaction center subunit, while *F*_A_ and *F*_B_ are located in an extrinsic protein that can be tightly or loosely bound (Scott et al. 1995; Nitschke et al. 1990a, b; Vassiliev et al. 2001; Heinnickel et al. 2005; Malkin 2006; Heinnickel et al. 2007; Jagannathan and Golbeck 2008; Romberger et al. 2010).

## Evolution of Type II reaction centers

**Fig. 3:**
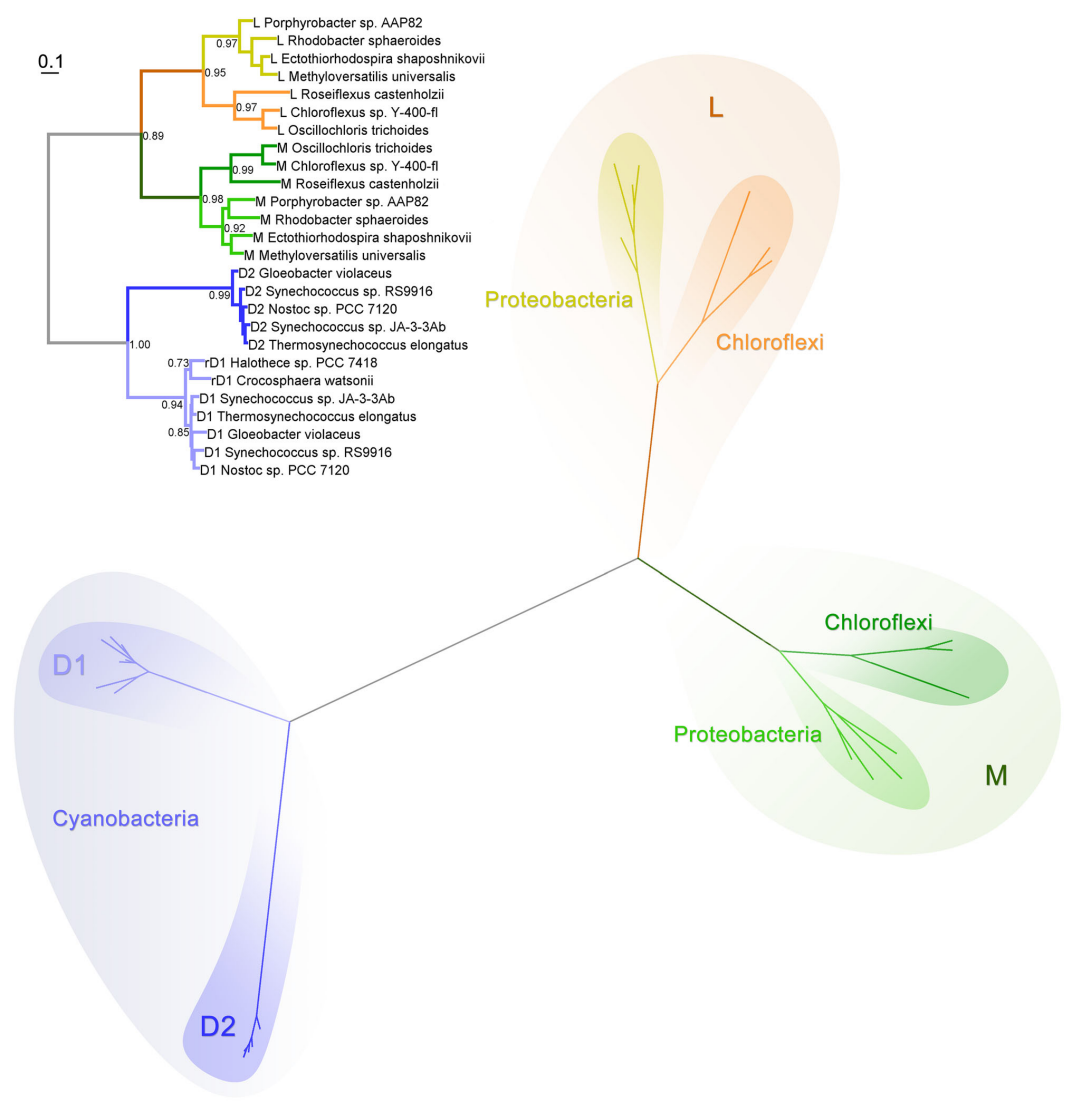
Maximum likelihood phylogenetic tree of Type II reaction center subunits. At the *top*, the tree is shown as a rectangular phylogram, and the same tree is displayed at the *bottom* as a radial phylogram to highlight the different clades. Sequences were aligned with ClustalX 2.1 (Larkin et al. 2007), and homologous positions were confirmed by overlapping the crystal structures of *B. viridis* (2PRC) and *T. vulcanus* (3ARC). The tree was calculated using PhyML 3.1 (Guindon et al. 2010), using the LG model of amino acid substitution and four substitution rate categories. The branch support was calculated with the Approximate Likelihood-Ratio Test (Anisimova and Gascuel 2006). The equilibrium frequencies, proportion of invariable sites, and the gamma shape parameter were set to be calculated by the program. Sequence alignments are available on request

All Type II reaction center proteins share a common origin. Therefore, it is possible to reconstruct their evolutionary relationships based on sequence alignments and phylogenetic analysis. The maximum likelihood phylogenetic tree in Fig. 3 shows the known evolutionary relationships between Type II reaction center proteins. It has basically the same topology as trees reported before (Beanland 1990; Blankenship 1992), which were constructed with a limited sequence data set and using parsimony-based phylogenetics. Similar results were obtained using structure-based phylogenies and distance methods (Sadekar et al. 2006). The tree indicates that Cyanobacterial Photosystem II reaction center proteins, D1 and D2, share a common ancestor, while the subunits from anoxygenic reaction centers, L and M, share a different common ancestor forming a separate branch of the tree (Beanland 1990; Blankenship 1992). It can be deduced that Type II reaction centers passed through a homodimeric stage before the evolution of heterodimericity. In other words, the heterodimeric character of all known Type II reaction centers evolved twice after two separate gene duplication events: one that produced the precursor genes for the L and M subunit, and the other for the D1 and D2 subunits.

Although the tree in Fig. 3 is technically unrooted, it can be assumed that the root is located at the divergence point between the D1/D2 branch and the L/M branch. This is because D1 and D2 share more sequence and structural similarity with each other, than either one of them with L or M. Within the L branch, the sequences from the Chloroflexales strains form one distinct group and the sequences from phototrophic Proteobacteria form another one. This can also be observed for the M branch (Fig. 3). Based solely on the molecular evolution of these reaction center proteins, there is no evidence for horizontal gene transfer of the *pufLM* genes from the phylum Proteobacteria to the Chloroflexi as suggested in Hohmann-Marriott and Blankenship (2011) or from the Chloroflexi to the Proteobacteria as suggested by Gupta (2003). This phylogenetic tree does not support the hypothesis that the reaction center genes in the phototrophic Chloroflexi or the Proteobacteria were acquired from a “protocyanobacterium” as suggested by Mulkidjanian et al. (2006) or that Cyanobacterial D1/D2 originated from L/M as implied by Xiong et al. (2000).

An important conclusion derived from this phylogenetic tree (Fig. 3), as was pointed out by Beanland (1990), is that D1 and D2 are as ancient as L and M subunits. What is more, the tree indicates that the D1/D2 lineage of reaction center proteins started evolving before L and M split into “Proteobacteria” and “Chloroflexi” types. This completely rules out the possibility that the phylum Cyanobacteria obtained their Type II reaction center proteins from a phototrophic member of the Chloroflexi or the Proteobacteria. In addition, there is no evidence in the phylogenetic tree to suggest that the ancestral forms of L and M subunits originated within the phylum Proteobacteria or the phylum Chloroflexi. It is instead more likely that the gene duplication events that led to the divergence of L from M and D1 from D2 are so ancient that they occurred before the Proteobacteria, the Chloroflexi, and the Cyanobacteria clades had time to evolve.

**Fig. 4:**
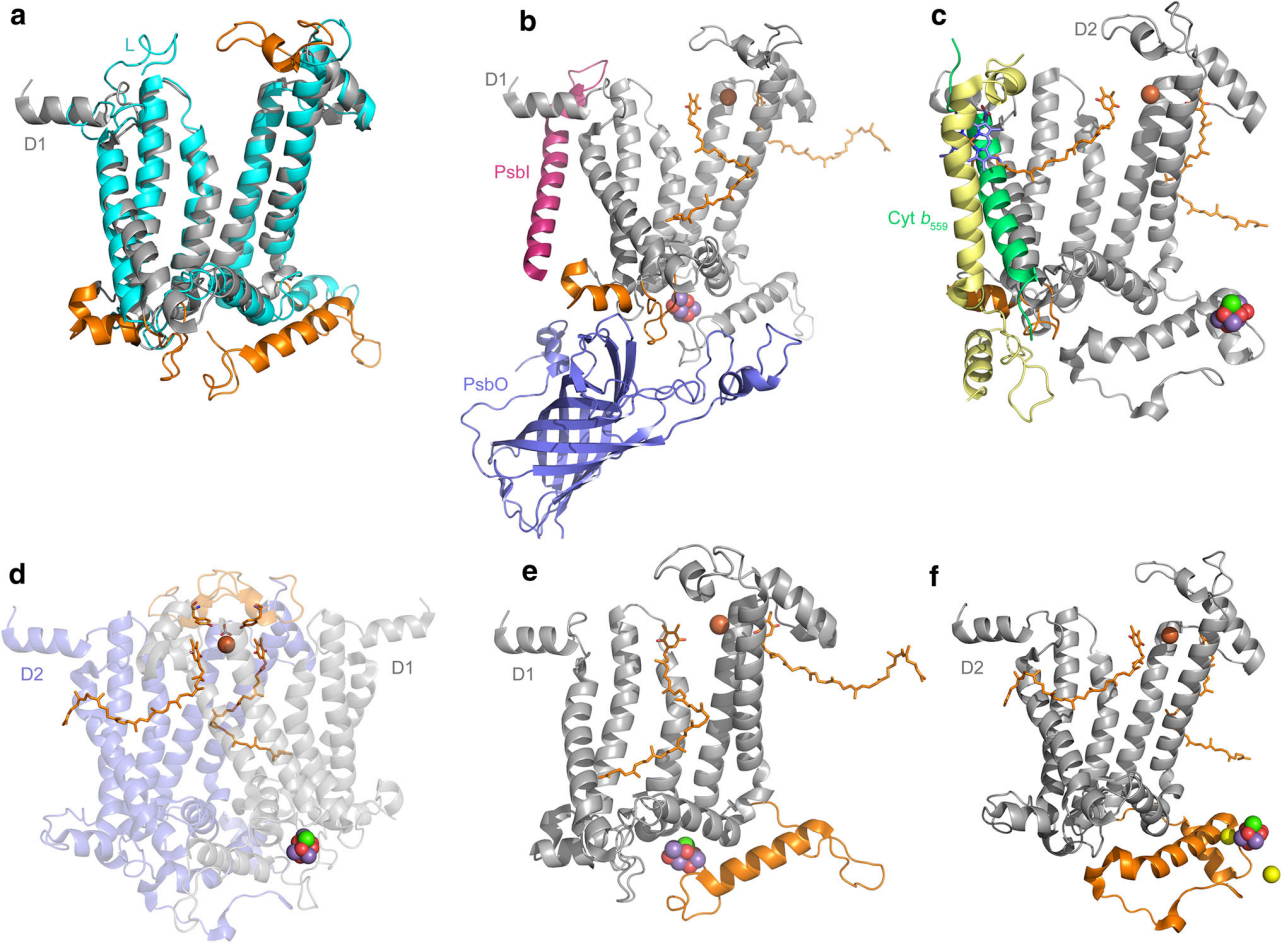
Structural comparisons of Type II reaction center proteins. **a** Overlap of D1 (*gray*) and L (*cyan*) subunits. Structural regions that are unique to D1 and D2 are highlighted in *orange*. **b**, **c** The interactions of ancillary subunits with a protein fold in D1 and D2 (*orange*). In D1, this region evolved to allow protein–protein interactions with the PsbI, PsbO, and CP43 subunits. In D2, it allows interactions with the Cytochrome *b*_559_ and PsbX. The presence of this fold in D1 and D2 suggests that before the evolution of oxygenic photosynthesis, the ancestral Photosystem II was already interacting with ancillary subunits. **d** A unique loop (*orange*) only present in D1 and D2. This region contains a tyrosine that coordinates the bicarbonate ligand of the non-heme Fe^2+^. **e**, **f** The C-terminal extension of D1 and D2 (*orange*) essential for the assembly and coordination of the Mn_4_CaO_5_ cluster. This C-terminal extension contains a parallel alpha helix in both subunits, suggesting that it was present in the ancestral Photosystem II before the D1 and D2 divergence

Despite D1 and D2 sharing only about 33 % sequence identity along the most conserved regions, they share not only remarkable structural similarities, but also features that distinguish them from their L/M cousins. There are three regions shared by D1 and D2 that are not present in L and M subunits (see Fig. 4a) (Ferreira et al. 2004; Umena et al. 2011). These are:

1. An extension and rearrangement of a loop that connects the first and second transmembrane helices. This region exists in D1 and D2 to connect the core proteins with accessory proteins (Fig. 4b). In D1, this region is in close contact with the PsbI subunit, the CP43 light-harvesting protein, and the PsbO extrinsic polypeptide. In D2, the region provides an interface with the Cytochrome *b*_559_ and the PsbX subunit (Fig. 4c). This implies that the ancestral homodimeric Photosystem II interacted with ancillary and peripheral subunits before the evolution of water oxidation.
2. An extension and rearrangement of a loop between the fourth and fifth transmembrane helix (Fig. 4d). The role of this extension is to provide a site of coordination to a bicarbonate molecule; it influences or modulates the electron transfer rate from *Q*_A_ to *Q*_B_ and provides proton pathways to the *Q*_B_ site (Petrouleas and Crofts 2005; Saito et al. 2013). The binding of bicarbonate at the acceptor side is a unique characteristic of Photosystem II, and it is not present in anoxygenic Type II reaction centers (Shevela et al. 2012).
3. A ~70 amino acid extension beyond the fifth transmembrane helix. In D1, this extension is essential for the binding of the Mn_4_CaO_5_ water splitting cluster (Fig. 4e). Four out of seven ligands to the metal cluster are within this extension (Ferreira et al. 2004; Umena et al. 2011). This extension also exists in D2 even though this subunit does not bind a cluster. The D2 extension, however, communicates with the cluster through the coordination of one of the Cl^−^ atoms (Fig. 4f) (Murray et al. 2008). These extensions also provide binding sites to the lumenal regions of the CP43 and CP47 subunits, and the extrinsic polypeptides.

**Fig. 5:**
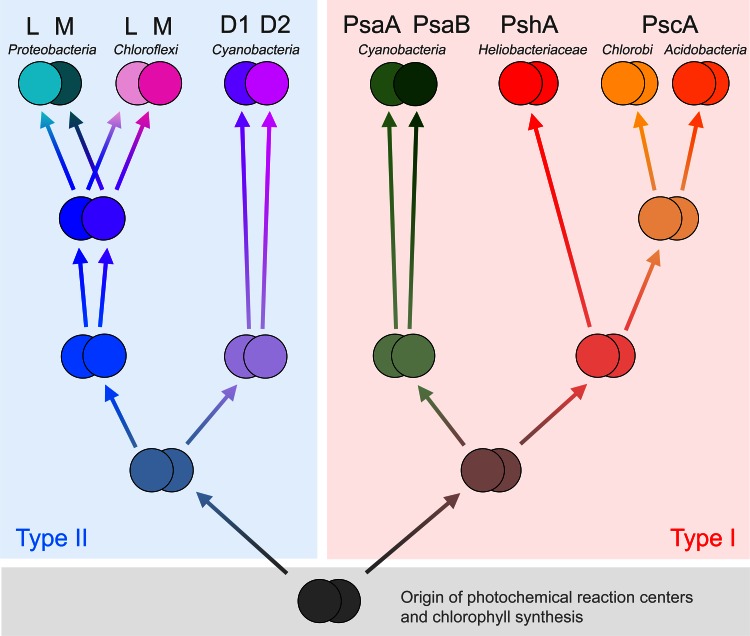
Evolutionary relationships of reaction center proteins as suggested by molecular phylogenies. At the beginning (*bottom*), the most ancestral photochemical reaction center protein was encoded by a single gene, this diverged giving rise to the precursor genes for the first Type II and Type I reaction center proteins. Initially both reaction centers were homodimeric. Type II reaction centers seem to have acquired heterodimericity by convergent evolution twice. Heterodimeric Type I reaction centers have only evolved once in the phylum Cyanobacteria

In conclusion, the evolution of Type II reaction center subunits can be summarized in three major steps (Fig. 5). First, the gene encoding the only subunit for an ancestral homodimeric Type II reaction center protein diverged into two forms: one was the ancestor of all L and M subunits, and the other was the ancestor of all D1 and D2 (Beanland 1990; Blankenship 1992; Rutherford and Nitschke 1996). This could have been speciation as an ancestral bacterium evolved into two different forms or a gene duplication event. Second, the ancestral gene of D1 and D2 duplicated, and each copy diverged and specialized in an ancestor to the phylum Cyanobacteria. Similarly, the ancestral genes to L and M duplicated in an ancestral bacterium that likely preceded the origin of the Chloroflexi and the Proteobacteria. And third, as the Chloroflexi and the Proteobacteria finally diverged, so did L and M in each group.

The nature of the ancestral bacteria in the first and second steps is hard to conceive, but it should never be forgotten that the branches in an evolutionary tree converge toward the root. Therefore, at some point in time, ‘the ancestor of the Cyanobacteria,’ ‘the ancestor of the Chloroflexi,’ and ‘the ancestor of the Proteobacteria’ were the same organism. In consequence and although it may seem improbable, the first two steps mentioned above could have happened in one and the same ancestral phototrophic bacterium. It is common in extant members of the Cyanobacteria, for example, that reaction center genes for Photosystem II and Photosystem I are found in multiple copies with different degrees of divergence within the same genome, as I will illustrate in the next section for D1. A similar situation could be envisioned for an ancestral phototrophic bacterium at the beginning of Type II reaction center evolution.

### Diversity of Cyanobacterial D1 proteins

One big difference between the Cyanobacteria and other phototrophic bacteria is that in many species some of the genes that encode reaction center subunits usually occur in multiple copies. For example, the genome of *Thermosynechococcus elongatus* BP-1 has three copies of the *psbA* gene encoding three distinct D1 proteins and two *psbD* genes encoding two identical D2 proteins. A more radical example is the genome of *Fischerella* sp. JSC-11, a true-branching filamentous heterocyst-forming cyanobacterium, which has six *psbA* genes encoding six distinct D1 proteins with different degrees of variation, ranging from 60 to 90 % sequence identity when compared to a D1 (PsbA1) from *T. elongatus*. *Fischerella* sp. JSC-11 also has two *psbD* genes encoding two different D2 subunits. The exact gene copy number varies from species to species, with the exception of some marine *Synechococcus* and *Prochlorococcus* strains, whose genomes carry only one copy of each gene.

In the case of the *psbA* genes encoding multiple D1 forms, five types of D1 proteins have been identified and could be categorized by their mode of expression and degree of sequence divergence:

1. The dominant form of D1. It is used under normal growth light and non-stressful laboratory conditions (Curtis and Haselkorn 1984; Golden et al. 1986). These include the D1 present in the crystal structures (Ferreira et al. 2004; Guskov et al. 2009; Umena et al. 2011).
2. The high-light form of D1. It is used under high-light or UV-light stress, and it is thought to have enhanced photoprotective properties (Clarke et al. 1993; Sugiura et al. 2010).
3. The microaerobic form of D1 or D1′. The expression of the gene encoding a D1′ is enhanced under low-oxygen concentrations (Summerfield et al. 2008; Sicora et al. 2009).
4. The dark D1, also known as “rogue” D1. The gene that encodes this isoform is expressed during the night or after prolonged darkness (Murray 2012; Toepel et al. 2008).
5. The far-red D1, also known as “super-rogue” D1 (Murray 2012). This type of D1 has been recently shown to be expressed after prolonged exposure to far-red light (Gan et al. 2014).

These additional copies and variant forms endow the bacterium with the ability to adapt to a wide range of environmental conditions. In general, two different photoprotective mechanisms have been described involving multiple copies of the *psbA* gene. The first mechanism consists in the stress-induced up-regulation of the expression of *psbA* genes encoding identical D1 proteins, and this should enhance the repair process to compensate for the increase in damage (Mulo et al. 2009, 2012). In the second mechanism, the dominant form of D1 is replaced by another D1 protein with particular amino acid changes that alter the functional properties of the Photosystem II complex (Sugiura and Boussac 2014). The best studied example of the second mechanism is the expression of D1 variants in which glutamate replaces glutamine at position 130 (Q130E). The glutamate provides a stronger H-bond to the pheophytin acceptor, Ph_D1_, making its redox potential more positive (Giorgi et al. 1996; Merry et al. 1998; Sugiura et al. 2010), and this should diminish the formation of chlorophyll triplets (Cser and Vass 2007; Sugiura et al. 2013).

In some species, a different *psbA* gene is shown to be transcribed under microaerobic conditions (Summerfield et al. 2008; Sicora et al. 2009); this type of D1 is usually known as D1′. A Photosystem II containing a D1′ (PsbA2) isolated from *T. elongatus* showed modifications in the proton-coupled electron transfer on specific steps of the enzyme cycle (Sugiura et al. 2012, 2013), and it seemed to copurify with a novel heme-containing protein (Boussac et al. 2013). It is still unclear what the physiological significance of such D1′-containing Photosystem II complexes is.

Murray (2012) pointed out that an extremely divergent form of D1 was present in the genome of some Cyanobacteria, different from the microaerobic D1′ variants. These were called “rogue” D1 (rD1) and are characterized by extensive modifications around the water-oxidizing complex and around the *Q*_B_ binding site, which may impair the binding of the Mn_4_CaO_5_ cluster and the exchangeable quinone, assuming that the protein is assembled into a Photosystem II complex. The rD1 sequences have about 65 % identity when compared to conventional D1 proteins. Based on phylogenetic analysis, Murray (2012) suggested that the rD1 variants as a group could represent a photosynthetic ‘missing link’ in the evolution of water oxidation.

The *psbA* gene encoding rD1 has been found to be transcribed in the N_2_-fixing *Cyanothece* sp. ATCC 51142, *Cyanothece* sp. PCC 7822, and *Crocosphaera watsonii* WH 8501 during the night, when oxygen sensitive enzymes such as nitrogenase or the uptake hydrogenase are active (Toepel et al. 2008; Shi et al. 2010; Zhang and Sherman 2012). In a recent transcriptomic analysis in the heterocystous cyanobacterium *Anabaena variabilis* TCC 29413, it was found that heterotrophic growth (darkness plus fructose) enhanced the expression of a *psbA* gene encoding a rD1 (Park et al. 2013). In the non-diazotroph *Acaryochloris marina* MBIC11017, a *psbA* gene for the rD1 was transcribed after prolonged exposure to dibromothymoquinone, an inhibitor of the Cytochrome *b*_6_ *f* complex under illumination (Kiss et al. 2012). It has been suggested that a rD1-containing Photosystem II may not be functional in water oxidation, but whether the mRNA is translated, and the proteins assembled into the complex or not have not been determined yet. Therefore, the exact role of this type of D1 is still unknown, but it may be relevant during the early hours of the morning.

In addition to these rD1 variants, another extremely different *psbA* gene was identified by Murray (2012) in *Synechococcus* sp. PCC 7335. This had a sequence identity of only ~55 % compared to any conventional D1. This D1 variant was labeled a “super-rogue” D1, and in the phylogenetic analysis by Murray (2012), the sequence was the earliest evolving D1. The new Cyanobacterial genomes have shown that these “super-rogue” D1 sequences are not unique to *Synechococcus* sp. PCC 7335 but are also found in a at least thirteen different strains, including *Chroococcidiopsis thermalis* PCC 7203, *Calothrix* sp. PCC 7507, and *Fischerella* sp. JSC-11, among others (Gan et al. 2014). These “super-rogue” D1 also show extensive modifications around the water-oxidizing complex and the *Q*_B_ site.

A recent study showed that the “super-rogue” D1 was located in a ~21-gene cluster containing multiple alternative reaction center subunits (Gan et al. 2014). The cluster contains not only the “super-rogue” D1 but also another standard D1 isoform, alternative versions of D2, CP47, and CP43. The gene cluster also contained alternative core subunits of Photosystem I and phycobilisome subunits. In addition, a red/far-red light-sensing phytochrome and two response regulators were also found in this cluster. Gan et al. (2014) demonstrated that during prolonged far-red light exposure, the gene cluster became up-regulated, and the entire photosynthetic apparatus was rebuilt with the alternative subunits. Most surprisingly, these strains synthesized chlorophyll *d* and *f*, which allowed the bacterium to grow photoautotrophically and perform oxygenic photosynthesis efficiently under far-red light. From the thirteen strains containing the “super-rogue” D1 and the gene cluster for the far-red acclimation response, seven have been tested positive for chlorophyll *f* synthesis (Gan et al. 2014).

Further investigation of the conditions under which these diverse forms of D1 are expressed, how they alter Photosystem II photochemistry, and how they originated and evolved, could reveal clues on the origin of water oxidation.

### The reaction center from *Roseiflexus*

The Type II reaction center from the sequenced *Roseiflexus* species, members of the phylum Chloroflexi, is unique among all reaction centers. Some of those particularities might be of relevance for the origin of oxygenic photosynthesis. Their Type II reaction centers are unusual because both subunits, L and M, are encoded in a single gene (Yamada et al. 2005). The gene in *R. castenholzii* DSM 13941 is predicted to encode a 641 amino acid protein and that in *Roseiflexus* sp. RS-1 one of 643 amino acids. The L subunit is located at the N-terminus, while the M subunit is encoded at the C-terminus. In between the L and M domains, there is a ~85 amino acid-long region that connects L with M, and this region is unique to *Roseiflexus* spp. Yamada et al. (2005) using hydrophobicity analysis found within this region an additional putative transmembrane helix, following immediately after the fifth helix of the L subunit domain. Therefore, the *pufLM* gene from *Roseiflexus* spp. encodes a Type II reaction center protein with eleven transmembrane helices, the first five are from the L subunit, a unique sixth helix connecting L with M, and the last five helices are from the M subunit.

**Fig. 6:**
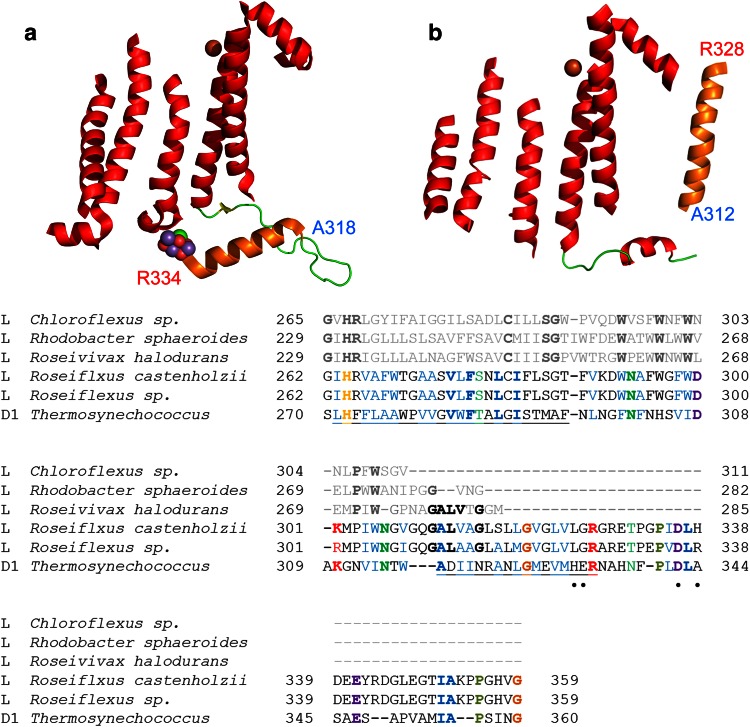
Sequence alignment of the L subunit from *Roseiflexus* spp. and the D1 subunit from *T. elongatus*. The alignment shows that the parallel alpha helix that in D1 is essential for the assembly and coordination of the Mn_4_CaO_5_ cluster **a** has sequence and structural homology to a putative sixth transmembrane helix predicted from the fused LM reaction center subunit in *Roseiflexus* (**b**). In *bold colored letters*, the conserved amino acids between the two *Roseiflexus* sequences and *T. elongatus* are highlighted. The *colored letters* that are *not in bold* show positive amino acid substitutions. The *underline* highlights the fifth transmembrane helix and the following alpha helix (parallel in D1 and transmembrane in L from *Roseiflexus* spp.). Sequences from a few phototrophic Chloroflexi and Proteobacteria strains are also shown. Surprisingly, the L subunit from the proteobacterium *Roseivivax halodurans* extends middle way through the predicted sixth helix. The *black dots* under the *T. elongatus* sequence are the ligands to the Mn_4_CaO_5_ cluster. **a** The transmembrane and parallel alpha helices of D1. **b** A homology model of the L subunit from *Roseiflexus* built using the subunit from *B. viridis* as a template. The model was made with the SWISS-MODEL automated service (Guex et al. 2009). The position of the putative sixth helix depicted in **b** is only hypothetical and just for illustration purposes

Although one protein is predicted from the gene sequence, isolation of the reaction center followed by denaturing SDS electrophoresis showed that the L and M subunits had been cleaved (Yamada et al. 2005; Collins et al. 2009), suggesting posttranslational processing of the original gene product at the C-terminus of the L subunit. In Photosystem II, a 9 to 16 amino acid extension at the C-terminus of the D1 protein is cleaved as part of the assembly process of the Photosystem II complex. This is required to allow full activation of the Mn_4_CaO_5_ cluster (Nixon et al. 1992), because the terminal carboxylate group of Ala344 provides a bidentate ligand to two of the Mn atoms (Umena et al. 2011). The posttranslational cleavage of the D1 protein and the cleavage of the *pufLM* gene product strike the author as too similar to be coincidental. To explore the possibility of an unexpected relationship between the Type II reaction center of *Roseiflexus* spp. and Photosystem II, and to try to understand the origin or role of the region between the L and M subunit, I took a closer look at the *Roseiflexus* spp. LM predicted sequence (see Fig. 6).

A sequence comparison of the C-terminus from *Roseiflexus* spp. L and the D1 from *T. elongatus* is shown in Fig. 6. The homology between the extension at the C-terminus of the L subunit and the C-terminus of D1 is remarkable. Moreover, the predicted sixth transmembrane helix in *Roseiflexus* in the L and M interconnecting region corresponds to the C-terminal alpha helix that in D1 is important for the coordination of the water-oxidizing complex (Fig. 6). This region also exists in D2 as I have described in the previous section (Fig. 4). A survey of L subunits from phototrophic proteobacteria showed that some bacteria of the genus *Roseivivax* also have an L subunit with sequence homology that overlaps with the predicted sixth transmembrane helix of *Roseiflexus* spp. This implies that such C-terminal extensions beyond the fifth transmembrane helix in L and D1/D2 might have originated in the ancestor of all Type II reaction center core proteins.

These observations are important for two reasons: first, the protein domains that in D1 are necessary for the binding of the water-oxidizing complex might have not arisen as a new invention in the phylum Cyanobacteria, from an interaction with catalase, for example (Raymond and Blankenship 2008), but from protein structures already present in the ancestral Type II reaction center. Second, the posttranslational processing of the C-terminus of D1 proteins in oxygenic photosynthesis and the posttranslational processing of the L/M precursor protein of *Roseiflexus* spp. may be homologous processes. This cleavage could have evolved from processing of the C-terminus of an ancestral Type II reaction center protein, a process that in the Cyanobacteria was later adapted to allow controlled activation of the Mn_4_CaO_5_ cluster.

## Evolution of Type I reaction centers

**Fig. 7:**
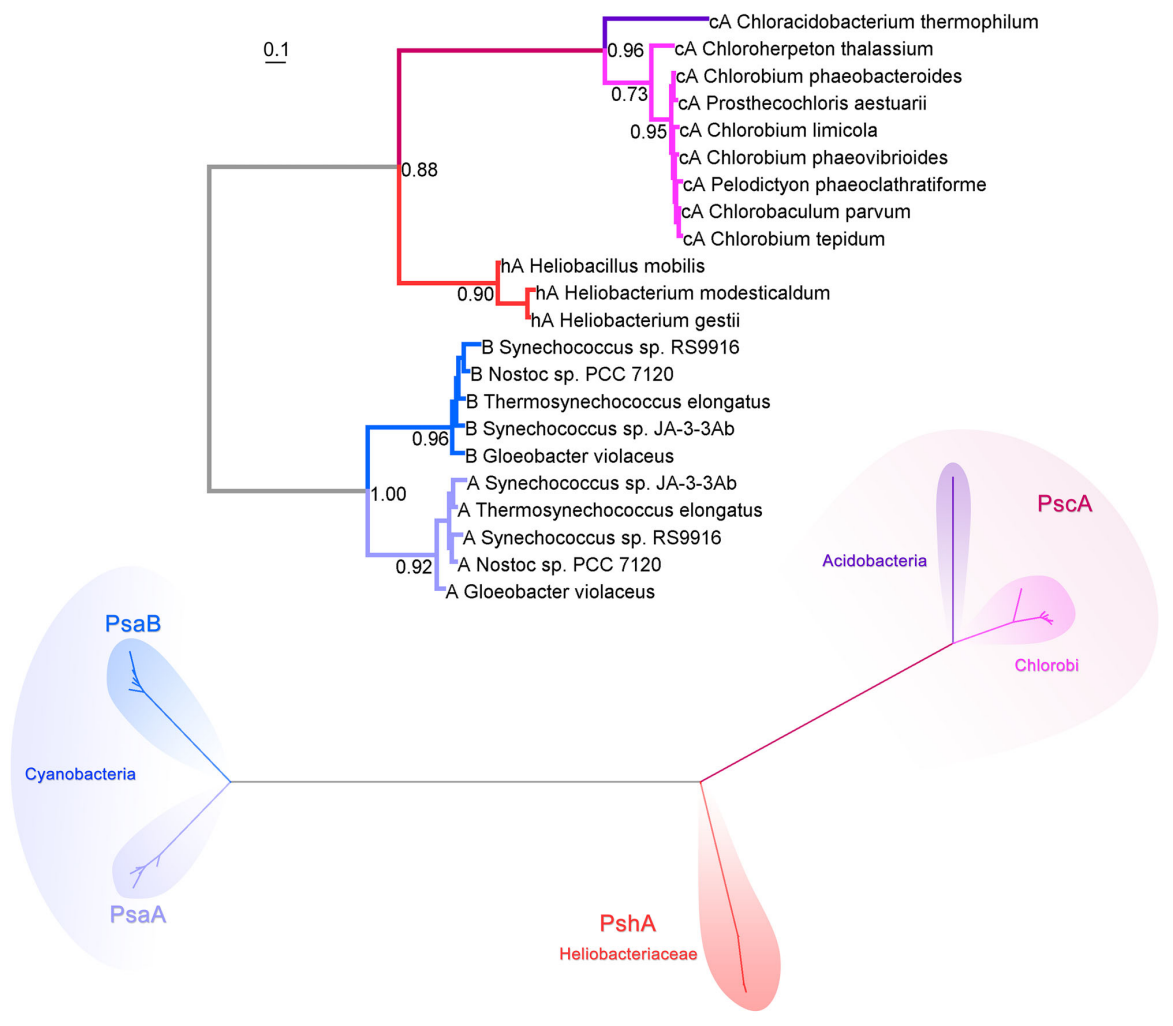
Maximum likelihood phylogenetic tree of Type I reaction center subunits. The tree was calculated using the same method as described in Fig. 3, and the sequence alignments are available on request. On *top*, the tree is shown as a rectangular phylogram and at the *bottom* in a radial form to highlight the different clades

The phylogenetic relationships of Type I reaction center core subunits calculated using maximum likelihood are shown in Fig. 7. It has basically the same topology as a tree reported by Bryant et al. (2007) using distance methods, which included for the first time the sequence from *C. thermophilum.* The tree is also similar to that constructed by Jagannathan et al. (2012) using in addition to distance, maximum likelihood and parsimony methods, and also see Sattley et al. (2008). These phylogenetic trees were built using alignments of the entire sequences of the reaction center subunit from all known Type I reaction centers. However, in contrast to Type II subunits, reconstructing the phylogeny of Type I reaction center proteins is slightly more difficult. This is mostly because there is a lower level of sequence homology among Type I reaction center subunits, especially when Photosystem I subunits PsaA and PsaB are compared with PscA and PshA. Thus, analyses with alternative sequence alignments have obtained different topologies for the phylogeny of Type I reaction centers (Rutherford et al. 1996; Mix et al. 2005; Hohmann-Marriott and Blankenship 2011).

The tree in Fig. 7 shows that the PscA protein present in Chlorobiales and in *C. thermophilum* is more similar to each other than to any other Type I reaction center protein. However, the PscA in *C. thermophilum* is distinct from that in the Chlorobiales strains and branches out before these diversified. The tree also shows that the PshA in heliobacteria is slightly more closely related to PscA than it is to PsaA and PsaB in Photosystem I. In other words, the ancestral protein to PsaA and PsaB in Photosystem I separated from the other Type I reaction center subunits, PshA and PscA, before these ones diversified into the Heliobacteriaceae and the Chlorobiales/Acidobacteria subgroups. Similar to the phylogeny of Type II reaction centers, there is no indication of horizontal transfer of Type I reaction center genes.

When the PscA subunit of *C. thermophilum* is compared to that in the Chlorobiales, the greatest homology is found between the eighth to the tenth transmembrane helix (2nd–4th in Type II core proteins) at 49 % identity as calculated using BLAST. This region of greater homology includes the *F*_X_ and special pair binding site. The antenna region, on the other hand, has a sequence identity of about 30 %. The PscA subunit from both phyla has a sequence identity of about 30 % to PshA in heliobacteria, encompassing the last seven transmembrane helices and excluding a large sequence extension present in the PscA subunit from *C. thermophilum* between the seventh and the eighth helix. The sequence identity between any PscA or PshA when compared to PsaA and PsaB of Photosystem I is of about 26–28 % and only including the region from the eighth to the tenth transmembrane helix. This is almost at the “twilight zone” of sequence homology. The sequence identity falls well below 25 % when the antenna domain of either PshA or PscA is compared to that of Photosystem I subunits. Even less homology is found when the antenna domains of any Type I reaction center protein are compared to the CP43 and CP47 subunits of Photosystem II. Such low sequence identity between the PsaA/PsaB family of reaction center proteins compared to PscA and PshA is reflected in the phylogenetic tree by the long branch that separates them (Fig. 7). Therefore, it is at this point of separation between the phylum Cyanobacteria and the anoxygenic phototrophic bacteria that the root of the phylogenetic tree is most likely to be placed. Rooting the Type I reaction center phylogeny at this point is in fact similar to the evolution of Type II reaction centers, where the ancestor of the D1 and D2 subunits of Photosystem II splits early from the ancestral L and M subunits (Fig. 5).

As it has been mentioned above and noted before (Rutherford et al. 1996; Jagannathan et al. 2012), the overall homology when comparing all Type I reaction center proteins from each group is generally very low. In particular, the antenna region shows greater divergence than the core domain, and only vestigial sequence homology, at best, can be retrieved from the first six transmembrane helices. Only a few histidine positions might be conserved as they provide ligands to (bacterio)chlorophyll molecules. A sequence comparison of the six transmembrane helices that make the antenna domain from all Type I reaction center proteins (excluding *C. thermophilum*) showed that only 4 out of 159 amino acid positions were strictly conserved across them all (Mix et al. 2005). That is 2.5 % overall identity of the transmembrane regions of the antenna domain. A phylogeny constructed from that sequence alignment showed a distorted tree with an extremely long branch for the PscA subunits (Mix et al. 2005), which is inconsistent with the greater sequence homology between PscA and PshA. Attempts to repeat the same phylogenetic analysis using the same data proved unsuccessful by a variety of methods due precisely to the low sequence homology. In that phylogenetic analysis, the root was suggested to be located at this very long branch, and thus the reaction center protein from the Chlorobiales was implied to be the earliest evolving one, with PsaA and PsaB sharing a more recent common ancestor with PshA (Mix et al. 2005).

A similar evolutionary tree showing PshA sequences grouping with PsaA and PsaB was presented by Hohmann-Marriott and Blankenship (2011). In this phylogenetic three, the reaction center core domain of the Type I subunits was aligned with Type II subunits. If that approach was possible it could prove ideal for the study of reaction center evolution and diversification, because it should provide a molecular basis for the Type I/Type II divergence. Unfortunately, the sequence alignment and methods to calculate the evolutionary tree for Type I and Type II reaction centers shown in Hohmann-Marriott and Blankenship (2011) were neither described nor discussed.

The evolution of Type I reaction center proteins can be summarized in a few steps based on the phylogeny of the available sequences (Fig. 5):

1. An ancestral Type I reaction center protein diversified into at least two forms. One was ancestral to all homodimeric anoxygenic Type I reaction center subunits. The second form was ancestral to the PsaA and PsaB subunits of Photosystem I; this ancestral Photosystem I was a homodimer, existing before the last common ancestor of the known diversity of the phylum Cyanobacteria.
2. The first of these proteins diversified into at least two other forms, one present in heliobacteria today. The second was ancestral to all PscA.
3. In a later stage, the ancestral PscA diversified into two more forms, one ancestral to the PscA found in Chlorobiales and a second one ancestral to that in *C. thermophilum* from the Acidobacteria.
4. Within the lineage that gave rise to the phylum Cyanobacteria, a duplication of the reaction center gene occurred leading to the evolution of a heterodimeric Type I reaction center.

### Diversity of PsaB subunits in Photosystem I

The Cyanobacteria are the only phylum of phototrophic bacteria where some species have multiple copies of Type I reaction center core subunits. The exact number of additional copies of the *psaA* and *psaB* genes depends on the species. For example, in the genome of *Nostoc punctiforme* or *Nostoc* sp. PCC 7120, there are only two copies of the *psaB* gene and only one copy of *psaA*. A more extreme case is the genome of *Chroococcidiopsis thermalis* or *Fischerella* sp. PCC 7414, where there are four different *psaB* genes and two different *psaA.* The notion that some species could also switch PsaB proteins to acclimate to different environments was only recognized recently with the availability of new genomes (Raymond and Blankenship 2006; Ekman et al. 2011; Park et al. 2013; Gan et al. 2014). The first experimental evidence came from a quantitative proteomic study of heterocysts from *N. punctiforme* (Ekman et al. 2011). Ekman et al. (2011) noted that in a knock-out mutant of the uptake hydrogenase, a second copy of the PsaB subunit was significantly less abundant in the heterocysts of the mutant than in the heterocysts of the wild-type strain. This alternative PsaB was named PsaB2. Magnuson et al. (2011) modeled the PsaB2 of *N. punctiforme* and noted that a few mutations clustered around the phylloquinone and *F*_X_ cofactors. These were proposed to tune their energetic properties and a specific tuning mechanism, and rationale was proposed by Rutherford et al. (2012). Transcriptomic studies showed that the alternative PsaB2 is transcribed both in vegetative cells and in heterocysts (Campbell et al. 2007; Park et al. 2013). However, the exact environmental conditions that could cause the preferential use of one PsaB isoform over the other have not been determined yet. The tuning mechanism suggested by Rutherford et al. (2012) predicts that a PsaB2-containing Photosystem I could be more efficient in anaerobic conditions. On the other hand, the well-characterized Photosystem I is adapted to function in the presence of O_2_ but at the expense of some efficiency. This model also provides an explanation for the heterodimeric nature of Photosystem I and implies that the PsaA and PsaB divergence occurred as a response to oxygenic photosynthesis, after the evolution of water oxidation by Photosystem II (Rutherford et al. 2012).

In the recent study by Gan et al. (2014), alternative versions of both PsaA and PsaB subunits were found in the same gene cluster required for red/far-red acclimation. The newly synthesized Photosystem I under far-red illumination contained chlorophyll *f* and had enhanced absorption above 700 nm in comparison with the standard Photosystem I. It should be noted that the alternative PsaB in the far-red acclimation response is phylogenetically distinct to that found in *N. punctiforme* and a few other Cyanobacteria as described in (Magnuson et al. 2011) (Cardona, T. unpublished results). Therefore, these two types of PsaB subunits represent separate gene duplication events, which might reflect two different acclimation strategies.

## The Type I and Type II divergence

Type I and Type II reaction centers share a common origin. However, how these two types of reaction centers diverged and specialized to perform different types of chemistry still remains controversial. Every possible evolutionary scenario has been proposed or implied. A popular hypothesis suggests that Type I reaction centers are more like the ancestral reaction center and gave rise to Type II reaction centers, see Barras (2013), for example. Less popular but still discussed is the alternative view where Type II gave rise to Type I reaction centers, see Larkum (2006), for example. Others have suggested that the ancestral reaction center was neither a Type I nor a Type II but had mixed characteristics. Some examples for evolutionary scenarios are listed below; they differ on which group and reaction center type are suggested to be more ancestral. This list is not meant to be exhaustive:

1. Type II reaction centers from phototrophic Chloroflexi (Cavalier-Smith 2006).
2. Type II reaction centers from phototrophic Proteobacteria (Xiong et al. 1998, 2000).
3. Type I reaction centers from heliobacteria (Gupta 2003, 2012).
4. Type I reaction centers from a ‘protocyanobacterium’ (Mulkidjanian et al. 2006; Mulkidjanian and Galperin 2013).
5. Type I reaction centers from phototrophic Chlorobi(Baymann et al. 2001).
6. ‘Type 1.5’ first or an ancestral reaction center with mixed characteristics (Rutherford et al. 1996; Allen 2005; Sadekar et al. 2006).

Other evolutionary scenarios have been proposed as well. Recently, it was suggested that a homodimeric Type I reaction center gave rise to heterodimeric Type I, and this evolved into Photosystem II and anoxygenic Type II reaction centers (Nelson 2013). Other proposals invoke horizontal gene transfer from heliobacteria into a proteobacterium to generate a primordial cyanobacterium with two reaction centers (Mathis 1990). Another possibility was considered by Björn and Govindjee (2009), and they suggested that the phylum Cyanobacteria could have derived from horizontal gene transfer of a Type II reaction center from the Chloroflexi to the Chlorobi. See also Olson and Blankenship (2004) for lengthier discussions on different evolution models.

The phylogenetic trees for Type II (Fig. 3) and Type I (Fig. 7) reaction centers unambiguously resolve the debate on the ancestry of reaction centers. It should be clear then that all Type II reaction center core subunits (D1, D2, L, M) originated from one ancestral protein that underwent several episodes of gene duplication. In other words, all Type II reaction center subunits share a more recent common ancestor with each other than with any Type I reaction center protein. In a similar manner, all Type I reaction center core subunits (PshA, PscA, PsaA, PsaB) also originated from an ancestral protein. Therefore, they share a more recent common ancestor with each other than with any Type II subunit. The ancestral Type II and the ancestral Type I reaction center proteins are two different proteins, but these two are indeed homologous and originated from an even older reaction center subunit (summarized in Fig. 5). In consequence, models that suggest Type II subunits evolved from a Type I subunit in the Chlorobiales, the Cyanobacteria, or the Heliobacteriaceae family of Firmicutes are not consistent with the phylogenetic data. Similarly, models that suggest Type I subunits originated from a Type II reaction center protein from a member of the Chloroflexi or the Proteobacteria are not consistent with the available data. These models are inconsistent with the data because the phylogenetic trees show that the divergence of the ancestral Type I and Type II reaction center proteins had to occur before the diversification of the known protein forms. It can be concluded then that the Type I/Type II split occurred before the diversification of the extant groups of phototrophic bacteria.

After the origin of the first photochemical reaction center protein, selective pressures led to the evolution of two distinct types of core proteins: one became the primordial Type I core subunit, and the other became the primordial Type II. Only at a later stage, each one of these diversified into the different subunits characteristic of each phyla of phototrophic bacteria (Fig. 5). Thus, models of reaction center evolution 1–5 in the list above (Type I first or Type II first) can be confidently ruled out. The evolutionary model described by Nelson (2013) can also be ruled out, because the data does not support any model where Type II originated form heterodimeric Type I reaction centers. Besides, any model that suggest Cyanobacteria got both reaction centers via horizontal gene transfer from one or more donors or that the group arose as the fusion of two bacteria, one containing a Type I and one containing a Type II can be ruled out too (Mathis 1990; Blankenship 1992). In almost two decades, since the first Cyanobacterial genome was sequenced (Kaneko et al. 1996), no evidence has been provided suggesting that the phylum originated of such fusion event; an event which, with all certainty, should have left a mark in their genomes. The sequence and structural comparisons suggest that there was an ancestral reaction center protein, which assembled into a homodimeric complex (Fig. 5). The nature of the very first reaction center is difficult to predict, but it may have had mixed traits from each type as proposed before (Rutherford et al. 1996; Allen 2005; Sadekar et al. 2006).

**Fig. 8:**
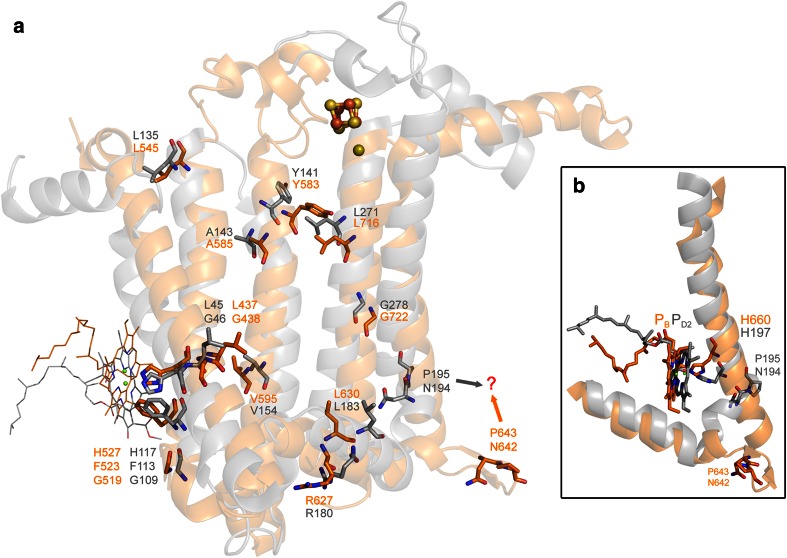
**a** Structural overlap of the D2 protein of Photosystem II (*gray*) and the last five transmembrane helices of the PsaB subunit of Photosystem I (*orange*). The amino acids that are strictly conserved are shown in *sticks*, and the corresponding sequence numbers are given according to the crystal structures 3ARC (Photosystem II) and 1JB0 (Photosystem I). **b** Position of the special pair pigment P_B_ and P_D2_ relative to the tenth and fourth transmembrane helices of PsaB and D2, respectively. The *question mark* highlights two residues that could be homologous but occupy different structural positions

Robust structural and sequence alignments of Type I and Type II reaction center subunits are required in order to reconstruct the properties of the ancestral reaction center at the dawn of photosynthesis. However, a proper alignment of Type I and Type II reaction center subunit sequences is by no means trivial (Rutherford et al. 1996), and it is impossible to obtain using conventional alignment algorithms such as Clustal or Muscle, regardless of the parameters used or amino acid substitution models applied. Indeed, the original arguments for Type I and Type II sharing a common ancestor reaction center came from structural and functional similarities (Nitschke and Rutherford 1991; Rutherford et al. 1996). This is because the sequence identity preserved between the two types of reaction centers is at best 5 %; needless to say, numerous insertions and deletions have occurred across sequences and lineages obscuring any sign of sequence homology. An overlap of the 3D structure of the D2 subunit of Photosystem II with the reaction center domain of PsaB of Photosystem I shows only 13 possible strictly conserved amino acids between the two, from approximately 350 (Fig. 8a, also see Sadekar et al. (2006) for a detailed structural analysis). The structural overlap can be used as templates to align the sequences and verify homologous regions. This reveals a number of interesting aspects about the evolution of reaction centers and the changes that had to occur from an ancestral reaction center to generate the two distinct types known.

The first interesting observation is a conserved histidine located in the second transmembrane helix of D1 and D2, which coordinates the peripheral chlorophylls, Chl_Z_ and Chl_D_, in Photosystem II (Figs. 1, 8a). This histidine is conserved in Photosystem I (eighth helix) and all Type I reaction center subunits (Baymann et al. 2001) with the exception of the PscA subunit from *C. thermophilum*, where it is a tryptophan. Judging by the almost identical positions of the chlorophylls, it is certain that these accessory chlorophylls were in the primordial reaction center too. Chl_Z_ and Chl_D_ in Photosystem II are necessary for efficient energy transfer from the CP43 and CP47 antenna proteins to the reaction center (Lince and Vermaas 1998; Vasil’ev and Bruce 2000). This could be the strongest argument so far to suggest that the primordial reaction center was closely associated with a light-harvesting complex. Based on the molecular phylogenies or structural comparisons of all reaction center proteins, it is not possible to determine yet whether the ancestral reaction center protein had an antenna domain or not.

The structural and sequence alignment also show that the loop that in Type I reaction center subunits binds *F*_X_, located between the eighth and the ninth transmembrane helix, was gained as an insertion in Type I or was lost in Type II as a sequence deletion. From the sequence and structural data, it is impossible to infer that the primordial reaction center contained a Fe_4_S_4_ cluster, as it has been suggested before (Allen 2005).

Arguably, the most interesting aspect about the divergence between Type I and Type II reaction centers is the fact that the histidine that coordinates the special pair of (bacterio)chlorophyll pigments is not conserved (Fig. 8b). Although the structural overlap shows that the pigments are almost at the same position, the histidine ligand in Type II and in Type I is provided from different places within the transmembrane helix (tenth in Type I and fourth in Type II). Both the structural and the sequence alignments argue against these histidines being at homologous positions (Margulies 1991; Schubert et al. 1998). In consequence, at some moment, a swap of histidine ligands to the special pair must have occurred.

## Diversification of bacteria

**Fig. 9:**
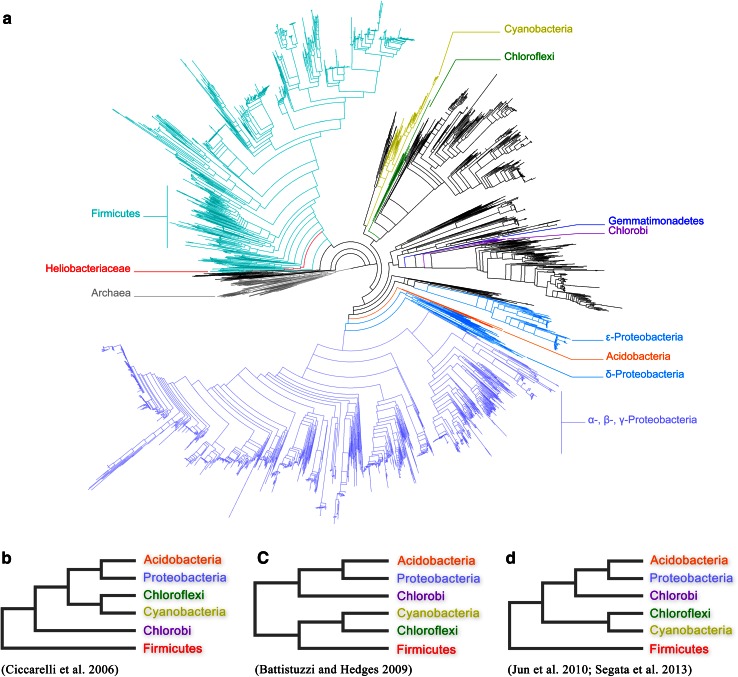
Evolutionary models depicting the positions of phyla with phototrophic bacteria. The tree shown in **a** was constructed by Segata et al. (2013) from hundreds of proteins and using thousands of genomes. The phylogenetic method, sequence alignments, and phylogenetic tree are freely available from the author’s website (http://huttenhower.sph.harvard.edu/phylophlan). The tree has been redrawn in here to highlight the relative positions of phyla with phototrophic bacteria. Notice the basal position of the Firmicutes, and the close relationships of the Cyanobacteria and the Chloroflexi, the Acidobacteria and the Proteobacteria, as well as the Chlorobi and the Gemmatimonadetes. **b**–**d** Schematic trees where all non-phototrophic clades have been omitted for simplicity. The tree in **b** is based on that reported by Ciccarelli et al. (2006), and the one in **c** is based on that reported by Battistuzzi and Hedges (2009). The tree in **d** is based on the work by Jun et al. (2010) and has a very similar branching pattern compared to that by Segata et al. (2013). However, while the latter is based on sequence alignments of protein sequences, the former was constructed using an alignment-free approach

Since the first molecular studies on the evolution of bacteria based on rRNA phylogenies (Woese 1987), our understanding of the relationships among the different bacteria groups has progressed enormously. Although many questions remain to be answered, a more consistent picture for the evolution of chlorophyll- and bacteriochlorophyll-based phototrophy is starting to emerge. Figure 9a shows a large phylogenetic tree for the evolution of prokaryotes constructed using >400 different proteins from among 3737 genomes (Segata et al. 2013). This tree includes for the first time sequences from all seven phyla known to contain phototrophic species. Figure 9b, c shows schematic representations of different phylogenetic trees constructed using very large datasets and different methodologies (Ciccarelli et al. 2006; Battistuzzi and Hedges 2009; Jun et al. 2010). Although they differ in a number of aspects, they have several important traits in common that are of relevance to understand the diversification of photochemical reaction centers.

The first notable characteristic is the close relationship of the Proteobacteria and the Acidobacteria. This close relationship had been noted before (Quaiser et al. 2003), and it is consistent throughout every evolutionary tree. In fact, in most cases, the phylum Acidobacteria clusters within the Proteobacteria as a sister group to the class Deltaproteobacteria but before the diversification of the phototrophic classes (Alpha-, Beta-, and Gammaproteobacteria). The evolution of proteins involved in the synthesis of chlorophyll, namely magnesium chelatase (BchIDH), magnesium protoporphyrin IX methyltransferase (BchM), and protochlorophyllide reductase (BchLNB), supports the close relationship of the acidobacterium *C. thermophilum* and the phylum Proteobacteria. Phylogenetic analyses (Bryant et al. 2012; Sousa et al. 2013; Bryant and Liu 2013) showed that the enzymes from *C. thermophilum* branch as sister group to the Proteobacteria, but most importantly, before their expansion and diversification. In other words, it is likely that these enzymes have been passed down by vertical descent from the last common ancestor of the Acidobacteria and the Proteobacteria. It can be deduced that the Acidobacteria and the Proteobacteria shared a common phototrophic ancestor. This poses interesting questions; did this ancestor have both Type I and Type II reaction centers? Did it have chlorosomes or the Fenna–Matthews–Olson complex as well?

The phylum Chlorobi always clusters within a large group that encompasses other non-phototrophic clades such as the Bacteroidetes and Fibrobacteres. Although the Chlorobi–Bacteroidetes–Fibrobacteres supergroup is distantly related to all other phyla of phototrophic bacteria, they seem to share a more recent ancestor with the Proteobacteria/Acidobacteria supergroup, than with the Chloroflexi/Cyanobacteria or the Firmicutes (Gupta 2003; Battistuzzi et al. 2004; Battistuzzi and Hedges 2009; Jun et al. 2010; Satoh et al. 2013; Segata et al. 2013), see Fig. 9. This is consistent with the PscA subunit in *C. thermophilum* and the Chlorobiales being more closely related, as we have discussed before (Fig. 7), and is an indication that the last common ancestor of these “supergroups” may have been phototrophic too.

Another conspicuous pattern that appears very consistently in large-scale phylogenetic analyses is the close relationship of the phylum Chloroflexi and the Cyanobacteria. In contrast, the evolution of proteins involved in chlorophyll synthesis shows very consistently that the enzymes found in the Chlorobiales and in the phototrophic Chloroflexi are more closely related, these include BchIDH, BchM, BchLNB, BchXYZ, and others (Xiong et al. 2000; Bryant et al. 2012; Sousa et al. 2013). In consequence, horizontal gene transfer has been proposed to explain these discrepancies (Boucher et al. 2003; Raymond 2009). However, these events of horizontal gene transfer are generally quite ambiguous as the direction of transfer is hard to determine.

Another aspect that seems to be shared by most of the phylogenetic trees is the basal position of the Firmicutes with regard to all other bacteria with photochemical reaction centers (Gupta 2003; Ciccarelli et al. 2006; Jun et al. 2010; Satoh et al. 2013; Segata et al. 2013). In some phylogenies, the Firmicutes branch as the earliest diverging group of all bacteria (Gupta 2003; Ciccarelli et al. 2006; Jun et al. 2010; David and Alm 2011; Satoh et al. 2013), while in others, they branch early but after the evolution of a few non-phototrophic clades (Jun et al. 2010; Segata et al. 2013). The only exception is the work by Battistuzzi and Hedges (2009) where the Firmicutes clustered in a larger group with the Cyanobacteria and the Chloroflexi. Interestingly, none of the proteins in the chlorophyll synthesis pathway from heliobacteria appear as the earliest branching in any of the phylogenetic studies available (Xiong et al. 2000; Bryant et al. 2012; Sousa et al. 2013). If chlorophyll synthesis had originated early in the evolution of the Firmicutes, this should be reflected in the phylogenetic trees, but this is not the case.

The last addition to the group of phototrophic bacteria is the phylum Gemmatimonadetes. The phylum was formally established by Zhang et al. (2003) based on 16S rRNA. This poorly studied group was placed in the vicinity of the Fibrobacteres (Zhang et al. 2003; Zeng et al. 2014). Curiously, the work by Segata et al. (2013) showed that the phylum Gemmatimonadetes is more closely related to the Chlorobi than to any other phyla containing phototrophic bacteria (see Fig. 9a). Zeng et al. (2014) proposed that *Gemmatimonas* sp. AP64, a strain of the phylum Gemmatimonadetes, obtained an entire photosynthesis gene cluster from a phototrophic proteobacterium in a single event of horizontal gene transfer. They also showed based on metagenomic data that other species within this phylum might be phototrophic. However, the phylogenetic trees presented in Zeng et al. (2014) for AcsF (Mg-protoporphyrin IX monomethyl ester cyclase), BchIDH, BchLNB, and PufLM suggest that the evolutionary history of this bacteria may be more complicated than expected. This is because even though the BchLNB and the PufLM cluster within the class Gammaproteobacteria, BchIDH and AcsF proteins do not seem to have the same origin. Both BchIDH and AcsF proteins did not branch within the Gammaproteobacteria but outside the Proteobacteria clade altogether. The close relationship of the Gemmatimonadetes with the Chlorobi, as estimated by Segata et al. (2013), may suggest that origin of phototrophic Gemmatimonadetes may have to be reevaluated when a better understanding of the diversity of the phylum is achieved.

## Resolving the discrepancies

In summary:

1. The Heliobacteriaceae family of Firmicutes branched early in the evolution of bacteria in comparison with the other phototrophic clades; however, the phylogenetic analyses of chlorophyll synthesis and reaction center proteins do not reflect this early divergence.
2. The large-scale phylogenetic analyses of all bacteria show consistently that the phylum Chloroflexi shares a more recent common ancestor with the Cyanobacteria than with any other phyla of phototrophs. Nevertheless, the Type II reaction center proteins of the phototrophic Chloroflexi are more closely related to that of the Proteobacteria, and the chlorophyll synthesis proteins are more closely related to that of the Chlorobiales.
3. The phylum Acidobacteria shares a more recent common ancestor with the Proteobacteria than with any other phototrophic group. This is reflected in the evolution of many chlorophyll synthesis proteins. However, the Proteobacteria and the Acidobacteria have different reaction centers and antenna systems.

The discrepancies among the phylogeny of reaction center proteins, the evolution of chlorophyll synthesis, and the phylogenetic relations of the phototrophic phyla are best explained if the following two criteria are met: first, the divergence of Type I and Type II reaction center proteins and the origin of chlorophyll synthesis occurred before the diversification of most groups of bacteria; and second, the last common ancestor of all phototrophs had both reaction centers.

The first criteria are clearly reflected in the phylogeny of Type I and Type II reaction centers, as their evolution suggests that the divergence of Type I from Type II reaction center proteins must have occurred before the diversification of the different clades of phototrophic bacteria. It is also reflected in the phylogeny of chlorophyll synthesis proteins, as it has been demonstrated that protochlorophyllide (BchLNB)and chlorophyllide reductases (BchXYZ) are the product of an ancient gene duplication (Raymond et al. 2004; Bryant et al. 2012; Sousa et al. 2013). Like Type I and Type II reaction center proteins, the phylogeny of BchLNB and BchXYZ protein sets suggests that their divergence must have occurred before the diversification of the extant groups of phototrophic bacteria. In other words, at the time of the last common phototrophic ancestor, the entire pathway for the synthesis of bacteriochlorophyll and at least two different types of reaction center proteins had already evolved.

These two criteria explain why Cyanobacteria have both types of reaction centers and the early divergence of D1/D2 and PsaA/PsaB proteins from their anoxygenic cousins. Hence, if members of the phylum Cyanobacteria are the only ones to have retained both reaction centers since the last common phototrophic ancestor; then, the reaction center proteins in this group (D1, D2, PsaA, PsaB) followed an independent evolutionary pathway from early on. This is consistent with the oxygenic subunits branching from the base of their respective phylogenetic trees (Figs. 3, 5, 7), with no indication of horizontal gene transfer. The recent discovery of the Melainabacteria, the non-photosynthetic Cyanobacteria, prompted Soo et al. (2014) to suggest that photosynthesis in the phylum Cyanobacteria might have been acquired relatively late by extensive horizontal gene transfer. Although it does look like water oxidation evolved after the Melainabacteria/Cyanobacteria divergence, it is more likely that the Melainabacteria lost their reaction centers. Perhaps it will not be too long before phototrophic Melainabacteria are discovered.

A phototrophic ancestor with two reaction centers also explains why phototrophic Chloroflexi have mixed traits from organisms that have Type I or Type II reaction centers (Bryant et al. 2012). It is also consistent with the phylum Cyanobacteria and the Chloroflexi sharing a common ancestor that harbored both reaction centers. Indeed, it was suggested recently that the Chloroflexi could have had a Type I reaction center that was later swapped with a Type II (Bryant et al. 2012), but they might just as well have had both. This is also applicable to the relationships of the Acidobacteria and the Proteobacteria. In fact, based on the large-scale phylogenetic analyses, the Acidobacteria and the Deltaproteobacteria could be defined as a separate phylum sister to the Alpha-, Beta-, and Gammaproteobacteria.

Finally, a primordial phototroph evolving before the diversification of the major bacterial phyla also explains why the PshA and chlorophyll synthesis proteins in heliobacteria do not appear as the most ancestral overall. This is because in spite of being one of the earliest forms of phototrophic bacteria known today, photochemical reaction centers did not originate within the heliobacteria. Reaction centers had already been evolving for a time before the Heliobacteriaceae family of phototrophic Firmicutes came into being.

## Concluding remarks

If the origin of photosynthesis dates back 3.8-3.5 Ga (Gigaannum, billion years) as is suggested by the geochemical record (Olson 2006; Allwood et al. 2006; Schopf 2011), this should almost immediately imply that the last ancestor of extant bacteria was a photosynthetic organism, as was proposed by Woese et al. (1985) and Woese (1987). There is very compelling geochemical evidence, based on sulfur isotope data and trace elements records for an early origin of oxygenic photosynthesis around 3.0 to 2.7 Ga ago, several hundred million years before the Great Oxygenation Event (Farquhar et al. 2011; Kasting 2013; Crowe et al. 2013; Lyons et al. 2014; Canfield 2014; Planavsky et al. 2014). The existence of Cyanobacteria in the late Archean implies that many of the phyla of bacteria had already appeared at the time. This is because the phylum Cyanobacteria is not particularly early evolving relative to the overall evolution of bacteria (Fig. 9). Consistent with this, David and Alm (2011) using an innovative phylogenomic approach calculated that most major phyla of bacteria originated and diversified during the late Archean, a period of rapid genetic innovation that peaked about 3.25 Ga ago. This rapid expansion was characterized by the birth of a quarter of all gene families, massive gene duplication, and horizontal gene transfer. It was followed by a process of massive gene loss peaking about 100 million years later. This is important because the earliest evolving redox genes during this Archean expansion were related to anoxygenic and oxygenic photosynthesis. It only makes sense if reaction centers and pigments evolved extremely early in the evolution of bacteria, if not before their last common ancestor, and then diversified as the numerous bacteria phyla expanded. It begs the question: could the invention of photosynthesis have triggered the explosion of bacterial diversity during the Archean?

Taking into account the very large phylogenetic distance between Type I and Type II reaction center proteins, between anoxygenic and oxygenic forms, and among all phyla of phototrophic bacteria; adding to these that photosynthesis could have been evolving for hundreds of millions of years before the expansion of bacteria, then it should be expected that many forms of photochemical reaction center evolved as bacteria diversified. Today’s diversity is only the tip of the iceberg of all the diversity that has existed since the origin of life. Accordingly, the known forms of phototrophy represent a very small subset of a much larger diversity that includes many reaction centers, antenna systems, and pigment forms. The rise of oxygen must have certainly played a role in the extinction of many phototrophic bacteria, and as opportunities for symbiotic relationships expanded with the evolution of eukaryotes, the dependency on phototrophy for survival declined. As the diversity of bacteria is mapped at a genomic level, new and unexpected phototrophs have been discovered (Bryant et al. 2007; Zeng et al. 2014). There are still countless forms of bacteria to be discovered and studied (Rappe and Giovannoni 2003; Rinke et al. 2013; Di Rienzi et al. 2013), and as our comprehension of bacterial diversity and evolution expands, so does our understanding of the natural history of photosynthesis.
